# Global Gene Expression Profiling Reveals Isorhamnetin Induces Hepatic-Lineage Specific Differentiation in Human Amniotic Epithelial Cells

**DOI:** 10.3389/fcell.2020.578036

**Published:** 2020-11-05

**Authors:** Yoshiaki Uchida, Farhana Ferdousi, Yun-Wen Zheng, Tatsuya Oda, Hiroko Isoda

**Affiliations:** ^1^School of Integrative and Global Majors (SIGMA), University of Tsukuba, Tsukuba, Japan; ^2^Alliance for Research on the Mediterranean and North Africa (ARENA), University of Tsukuba, Tsukuba, Japan; ^3^AIST-University of Tsukuba Open Innovation Laboratory for Food and Medicinal Resource Engineering (FoodMed-OIL), AIST, University of Tsukuba, Tsukuba, Japan; ^4^Department of Gastrointestinal and Hepato-Biliary-Pancreatic Surgery, Faculty of Medicine, University of Tsukuba, Tsukuba, Japan; ^5^Faculty of Life and Environmental Sciences, University of Tsukuba, Tsukuba, Japan

**Keywords:** human amnion epithelial cell, isorhamnetin, hepatic-lineage-specific differentiation, microarray and bioinformatics, natural compound

## Abstract

Human amnion epithelial cells (hAECs), derived from discarded term placenta, is anticipated as a new stem cell resource because of their advantages over embryonic stem cells (ESCs) and induced pluripotent stem cells (iPSCs), such as no risk of tumorigenicity and minimal ethical issue. hAECs have been reported to differentiate into hepatic-like cells (HLCs) with variable functionalities suitable for cell-based therapy of end-stage liver diseases, drug screening, and drug toxicity tests. On the other hand, a new research stream has been evolving to use natural compounds as stimulants of stem cell differentiation because of their high availability and minimum side effects. Isorhamnetin is a naturally occurring flavonoid commonly found in fruits and vegetables and has been reported to improve hepatic fibrosis and steatosis. In this present study, we have screened the differentiation potential of isorhamnetin in hAECs. The cells were grown on 3D cell culture and were treated with 20 μM of synthesized isorhamnetin for 10 days without adding any additional growth factors. DNA microarray global gene expression analysis was conducted for differentially expressed genes between isorhamnetin-treated and untreated control cells, gene expression validation was carried out using RT-qPCR method, and finally, several hepatic functions were assessed. Microarray analysis showed that isorhamnetin could activate essential biological processes, molecular functions, and signaling pathways for hepatic differentiation. Hepatic progenitor markers, *EPCAM* and *DLK1*, were upregulated in the isorhamnetin-treated hAECs. *AFP* was downregulated, while *ALB* was upregulated on Day 10. Furthermore, isorhamnetin-treated cells could show increased CYP enzyme mRNA levels, ICG uptake and release, glycogen storage activity, and urea secretion. Additionally, isorhamnetin-treated cells did not show any trace of transdifferentiation evident by significant downregulation of several colon- and cholangiocyte-specific markers. However, longer treatment with isorhamnetin did not promote hepatic maturation. Altogether, our findings indicate that isorhamnetin has a promising effect on directing the hepatic-lineage specific differentiation in hAECs.

## Introduction

Human amniotic epithelial cells (hAECs) are obtained from discarded term placenta, which is a medical waste product. hAECs are originated from the epiblast, thus retain pluripotent stem cell characteristics and give rise to all kinds of cells of three germ layers (Tamagawa et al., 2004; Miki et al., 2005; García-Castro et al., 2015). Besides, hAECs possess immune-privileged characteristics as they express all classical human leukocyte antigen (HLA) class I molecules as well as HLA-G (Adinolfi et al., 1982; Hammer et al., 1997), but do not express HLA class II molecules (Banas et al., 2008; Murphy et al., 2010). Moreover, hAECs do not possess telomerase and have no or low possibility of tumorigenicity (Miki et al., 2005; Bilic et al., 2008). hAECs have several other advantages over embryonic stem cells (ESCs) and induced pluripotent stem cells (iPSCs) such as easy isolation, reduced or no risk of rejection, and minimal ethical issues. Therefore, hAEC is anticipated as a new candidate for stem cell resource (Hori et al., 2006; Miki and Strom, 2006; Miki, 2011).

To date, several studies have shown that upon appropriate differentiation protocol, hAECs can be differentiated into cardiomyocytes, pancreas and lung epithelium, bone and fat cells as well as neural cells (Miki et al., 2005, 2010; Toda et al., 2007; Parolini et al., 2008; Murphy et al., 2010; Fang et al., 2012). Furthermore, studies have reported hepatic differentiation potentials of hAECs using multistep proliferation and differentiation protocols involving several combinations of growth factors and cytokines (Marongiu et al., 2011; Lin et al., 2015; Liu et al., 2018; Maymó et al., 2018). In our previous study, we have reported that the hAECs isolated from the adherent subpopulations of passaged primary cells have widely expressed stemness markers (Furuya et al., 2019). We have also shown that hAECs cultured in a 3D microenvironment as spheroids have highly expressed the stemness-related genes compared to their 2D counterpart (Ferdousi et al., 2019). In 3D culture of hAECs, we could generate functional hepatic organoids using several growth factors (Furuya et al., 2019).

In this context, a new research stream has been evolving to use medicinal plant extracts as stimulants of stem cells because of their high availability, low toxicity, and minimum side effects (Udalamaththa et al., 2016; Kornicka et al., 2017; Udagama and Udalamaththa, 2018; Saud et al., 2019). However, despite the fact that hAECs were discovered decades ago, few researches have been done on using natural compounds to optimize the microenvironment or to regulate the early biological events for controlled differentiation of hAECs. In our previous studies, we have reported that a natural compound rosmarinic acid could induce neuronal differentiation in hAEC (Ferdousi et al., 2019), whereas verbenalin-treated hAECs showed therapeutic potential for Alzheimer’s disease (Ferdousi et al., 2020). Another recent study has reported that vitamin C, a natural antioxidant, could promote the proliferation, migration, and self-renewal of hAECs *in vitro* and elevate the therapeutic potential of hAECs in premature ovarian insufficiency model mice (Hou et al., 2020). However, no study to date reports a natural bioactive compound manipulates the molecular fate and directs hepatic lineage-specific differentiation in hAEC.

Isorhamnetin (3-Methylquercetin or 3′-Methoxyquercetin) is a flavonoid naturally occurring in several fruits and plant-derived foods. Several studies have reported its preventive effects against metabolic disorders, specifically against liver diseases (Lee et al., 2010; Yang et al., 2016; Zhang et al., 2016; Ganbold et al., 2019; Liu et al., 2019). Isorhamnetin exerts its bioactivities through regulating Wingless-related integration site (Wnt)/β catenin and transforming growth factor-beta (TGFβ) signaling pathways. These pathways are implicated in almost every facet of liver biology- from liver cell fate decision to liver organogenesis and to liver pathologies (Clotman et al., 2005; Clotman and Lemaigre, 2006; Nejak-Bowen and Monga, 2008; Touboul et al., 2016; Russell and Monga, 2018).

In this perspective, we have hypothesized that isorhamnetin may have the potential to induce directed differentiation of hAECs toward hepatic lineage. In the present study, we have investigated the early biological events regulated by isorhamnetin to induce hepatic-lineage-specific differentiation of hAECs maintained in 3D culture conditions through gene expression profiling and further validated several hepatic function tests.

## Materials and Methods

### Extraction of Amnion Epithelial Cells

In our previous studies, a detailed methodology of hAEC extraction, culture, and 3D spheroid formation have been documented (Ferdousi et al., 2019; Furuya et al., 2019). Briefly, hAECs were extracted from the term placenta. The amnion was aseptically separated from the chorion and washed with Hank’s Basic Salt Solution (CMF-HBSS). Pre-digestion buffer (CMF-HBSS, 0.5 mM EGTA) was added and then incubated at 37°C for 10 min. The pre-digestion buffer was discarded and 0.05% trypsin-EDTA was added and incubated for 40 min at 37°C. Two volume of DMEM was added and centrifuged at 200 × *g* for 10 min at 4°C. The supernatant was discarded, and the pellet was resuspended in DMEM.

### Cell Culture Maintenance

The cells were cultured in the Placental Epithelial Cell Basal Medium that contains no growth factor (Promo Cell, Cat. #C-26140). The medium was changed every 2 days. In order to subculture, cells were firstly washed with PBS and then the pre-digestion buffer was added. Cells were then incubated at 37°C for 5 min. Pre-digestion buffer was discarded and 0.05% trypsin-EDTA was added and incubated for 10 min at 37°C. Two volume of DMEM was added and centrifuged at 200 × *g* for 10 min at 4°C. After centrifugation, the supernatant was discarded, and the cell concentration was adjusted with a new medium.

### Preparation of 3D Cell Plate

To prepare a 3D cell culture plate, 400 μl of Lipidure solution was placed into each well of the 3D culture plate (Elplasia, Kuraray Co., Ltd.) and was sit for 2 min. And then, the Lipidure solution was aspirated out, to dry the plate. After drying, PBS was placed in each well, and the plate was centrifuged at 2000 × *g* for 15 min at room temperature. The PBS was then discarded, and the wells were washed twice with PBS. The plate was in an incubator until use.

### Spheroid Formation and Compound Treatment on 3D Cell Culture

The cells were cultured in a 3D plate and maintained in the Placental Epithelial Cell Basal Medium (Promo Cell, Cat. #C-26140). Spheroid was formed by seeding 8 × 10^5^ cells. After 24 h, the medium was changed with isorhamnetin (20 μM). The medium was changed every 2 days, and the cells were maintained for 10–20 days.

### RNA Extraction

Total RNA was extracted using 1 ml of ISOGEN (Nippon Gene, Japan). Chloroform (Wako, Japan) was added to obtain the supernatant aqueous phase. Then isopropanol (Sigma) was added at 0.8 volume, incubated for 5 min at 26°C (room temperature). The supernatant was discarded, and 1 ml of 70% EtOH was added and centrifuged (4°C, 12,000 × *g*, 5 min). The supernatant was discarded, and the RNA was dried at 26°C (room temperature) until no bubble was visible. Finally, the RNA solution was dissolved with Tris-EDTA buffer solution pH 8.0 (Sigma) and was quantified by using a NanoDrop 2000 spectrophotometer (Thermo Fisher Scientific, United States).

### Real-Time PCR

Complementary DNA synthesis was performed by using the Thermo Scientific Revert Aid First Strand cDNA Synthesis Kit (Cat. #K1691) following the manufacturer’s instructions. RT-qPCR was performed with the Superscript III reverse transcriptase kit (Invitrogen, United States) and 2720 Thermal cycler (Applied Biosystems, United States). For the quantification of amounts of transcripts, the TaqMan real-time RT-PCR amplification reactions were performed with a 7500 Fast Real-Time PCR system. TaqMan Universal PCR mix was used for the thermal cycling, and the run method was 95°C for 10 min, followed by 60 cycles of PCR (95°C for 15 s and 60°C for 1 min).

### Mitochondrial-Dependent Reduction of 3-(4,5-Dimethylthiazol-2-yl)-2,5-Diphenyltetrazolium Bromide (MTT) Assay

The cell viability was analyzed by using MTT assay to check the effects isorhamnetin on cytotoxicity. hAECs were seeded at 2 × 10^5^ cells/mL and incubated for 24 h. Cells were then treated with several concentrations of isorhamnetin for 72 h. After the treatment, MTT was added and incubated for 6 h at 37°C in the dark. After incubation, 10% sodium dodecyl sulfate (SDS) was added (100 μl) and incubated for over-night at 37°C. The optical density (OD) was measured at 570 nm with a microplate reader Varioskan LUX (Thermo Scientific, Rockford, IL, United States).

### Affymetrix Microarray Gene Expression

We conducted Affymetrix microarray gene expression profiling using GeneChip 3′ Expression Arrays and 3′ IVT PLUS Reagent Kit (Affymetrix Inc., Santa Clara, CA, United States). We used 250 ng of total RNA from each sample to generate amplified and biotinylated cRNA from poly (A) RNA in a total RNA sample following the users’ manual. For hybridization, 9.4 μG of cRNA samples were used. Human Genome U219 array strips (HG-U219) were hybridized for 16 h in a 45°C incubator, washed and stained. Imaging was conducted in the GeneAtlas Fluidics and Imaging Station. Each HG-U219 array strip is comprised of more than 530,000 probes, which cover approximately 36,000 transcripts and variants and represent more than 20,000 unique genes. The microarray was performed on two biological replicates of D0 control cells, three biological replicates of D10 control and D10 isorhamnetin-treated hAECs each, and two biological replicates of D20 control and D20 isorhamnetin-treated hAECs each. All data are deposited in a public functional genomics data repository, Gene Expression Omnibus (GEO) (Barrett et al., 2012), under the accession number GSE148777^1^.

### Microarray Data Processing and Analysis

Expression Console Software (provided by the Affymetrix) was used to normalize the raw data following the robust multichip average (RMA) algorithm^2^. Subsequent analysis of the gene expression data was carried out in the freely available Transcriptome Analysis Console (TAC) version 4 (Affymetrix, Japan). Differentially expressed genes (DEGs) were defined as the genes that satisfy both *p*-value < 0.05 (one-way between-subjects ANOVA) and fold-change (in linear space) ≥2 criteria simultaneously. The Molecular Signatures Database (MSigDB, v7.1) of Gene Set Enrichment Analysis (GSEA) software was used to determine significantly enriched Hallmark gene sets and gene ontologies (GO)^3^. MSigDB is a collection of annotated gene sets and one of the most widely used databases for performing gene set enrichment analysis (Subramanian et al., 2005; Liberzon et al., 2011, 2015). For gene functional annotation and pathway analysis, we also used an online data mining tool Database for Annotation, Visualization and Integrated Discovery (DAVID) v6.8. For any given gene list, DAVID tools are able to identify enriched biological GO terms (biological process, molecular function, and cellular component) and can discover enriched functional-related gene groups (Huang et al., 2008; Sherman et al., 2008). Heat maps were generated using a visualization software Morpheus^4^. Venn diagram was created using an online tool^5^. All data generated or analyzed during this study are included in this published article and its Supplementary Material.

### Indocyanine Green (ICG) Uptake and Clearance

Indocyanine green (Tokyo Chemical Industry Co., Ltd.) was dissolved to 5 mg/ml in DMSO as the stock solution and was diluted in culture medium to 1 mg/ml as the working solution. After 10 days of treatment on the 3D cell plate, cells were transplanted in a tube, and ICG was added. Then the cells were incubated for 30 min at 37°C. After that, cells were washed three times with PBS and added culture medium. At this time, ICG uptake was detected by light microscopy. ICG clearance was detected after 6 h following previous protocols (Lin et al., 2015; Furuya et al., 2019).

### Periodic Acid-Schiff (PAS) Staining

Periodic acid-schiff stains the stored glycogen in cells. At first, cells were washed with water. And then formalin was added for 2 min. Cells were washed with water, and 0.5% periodic acid was added for 7 min, after this, incubated in Schiff’s reagent for 10 min.

### Urea Assay

The concentration of urea in culture media was measured using the colorimetric assay according to the manufacturer’s instructions (640-1, Sigma, Japan). To obtain the final absorbance value for determining urea concentrations from the standard curve, the absorbance of the basal medium was subtracted from the absorbance of each test sample.

### Statistical Analysis

Comparisons between treatment groups were conducted using one-way ANOVA followed by Tukey’s *post hoc* test. Data were presented as means ± standard deviation (SD); unless otherwise mentioned. *P* < 0.05 was considered as significant. All analyses were performed using GraphPad Prism 8 software (GraphPad Software Inc., San Diego, CA, United States).

### Ethics Statement

The Ethical Review Committee of the University of Tsukuba Hospital approved the protocol. Informed written consent was received from the mothers who donated the placenta after delivery.

## Results

### 3D Cell Culture Enhanced the Pluripotency and Was Optimal Cell Culture for hAEC Differentiation

Recently, 3D cell culture has been widely used, especially in stem cell research, as it can closely mimic the microenvironment of our body and improve the differentiation. It was reported that cell culturing leads hAECs to lose their pluripotency (Ferdousi et al., 2019; Furuya et al., 2019). Thus, we first evaluated the effect of cell culture (2D or 3D) conditions on hAEC pluripotency. We found that several pluripotency markers, namely, Nanog Homeobox (*NANOG*), Octamer-binding transcription factor 4 *(OCT4)* and Sry-related HMG box gene 2 *(SOX2)*, were significantly upregulated in 3D cell culture compared to 2D culture after 10 days (Supplementary Figure 1A). Thus, 3D cell culture was considered optimal for the hAEC differentiation in our research.

### 20 μM of Isorhamnetin Was Determined as the Optimal Concentration for hAEC Differentiation

Next, the MTT assay was performed to decide the optimal concentration of isorhamnetin suitable for hAECs differentiation. From MTT assay, we found that up to the concentration of 10 μM, the number of viable cells was slightly increased (Supplementary Figure 1A). At the concentration of 20 μM, viable cell numbers on MTT significantly decreased. Therefore, firstly we evaluated gene expression changes in hAECs through microarray and RT-qPCR treated with 5, 10, and 20 μM of isorhamnetin. However, no significant changes in gene expression levels of hepatic progenitor markers were observed in lower doses (data not shown). Treatment with 20 μM isorhamnetin was found to be optimal to induce differentiation in hAECs.

### Isorhamnetin Regulated Gene Expression in hAECs on Day 10 (D10) Culture

**FIGURE 1:**
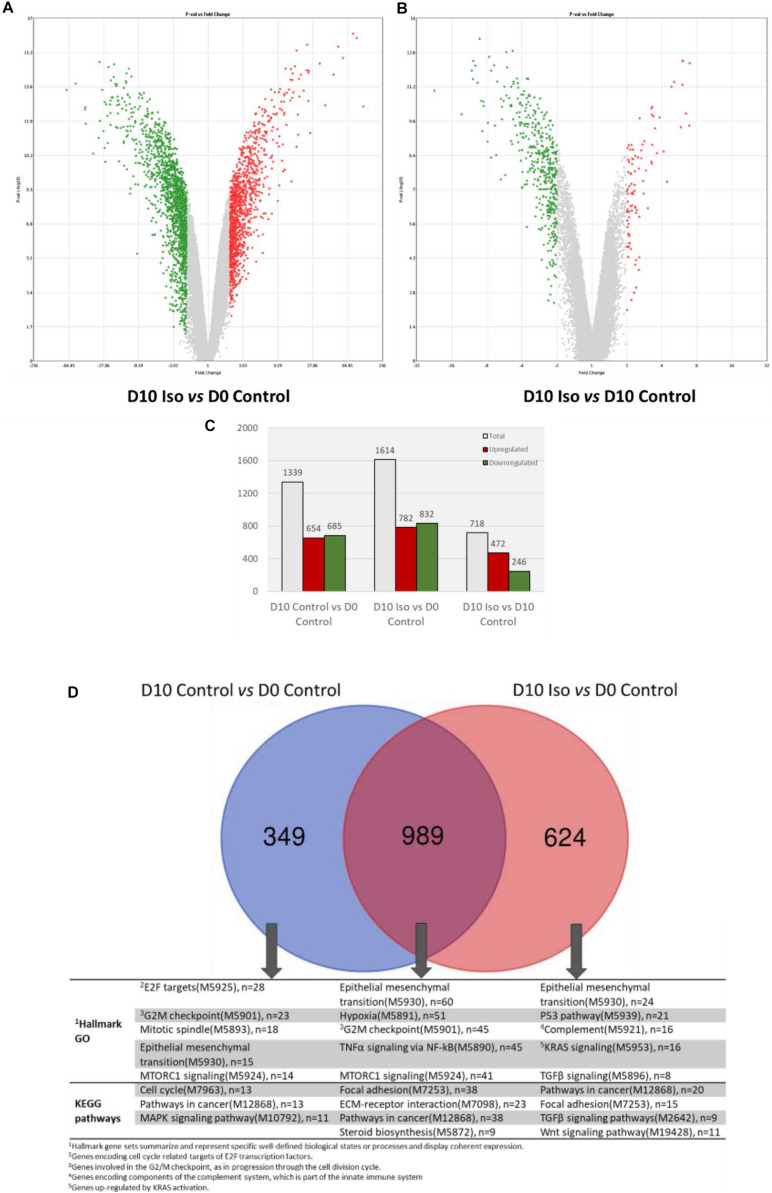
Volcano plots showing DEGs (fold change >2, *p* value < 0.05) between **(A)** D10 isorhamnetin-treated and D0 control hAECs, and **(B)** D10 isorhamnetin-treated and D10 control hAECs. The *Y*-axis corresponds to –log10 *p*-value, and the *X*-axis displays linear fold change. The red dots represent the upregulated genes; the green dots represent the downregulated genes. **(C)** Bar graph showing number of DEGs in each treatment pair. **(D)** Venn diagram showing common and unique sets of DEGs between each exposure. Blue circle denotes DEGs between D10 and D0 controls; red circle denotes DEGs between D10 isorhamnetin-treated and D0 control hAECs. Iso, Isorhamnetin.

Figures 1A,B show the DEGs in D10 isorhamnetin-treated hAECs compared to D0 and D10 control hAECs, respectively. A total of 1614 genes were differentially expressed in D10 isorhamnetin-treated hAECs compared to D0 control cells, among which 782 DEGs were upregulated, and 832 DEGs were downregulated (Figure 1C). When compared to D10 control hAECs, total 718 genes were differentially expressed in D10 isorhamnetin-treated cells, of which 472 were upregulated and 246 were downregulated. However, longer treatment with isorhamnetin for 20 days did not show much effect on the gene expressions (Supplementary Figures 2A–E). Therefore, we evaluated the effect of isorhamnetin on hAECs on D10 culture.

Figure 1D shows the common and unique genes between D10 control and isorhamnetin-treated cells. Although cells were maintained in Placental Basal Medium, the control cells showed changes in gene expressions related to cell cycle and epithelial-mesenchymal transition (EMT). As reported earlier, hAECs are primarily committed to ectodermal lineage-derived cells (Pan et al., 2006) and can actually express neuronal markers without any induction. Among the DEGs, 989 were commonly regulated in both control and treatment cells. Common functions include EMT, cell division, cell adhesion, and as well as tumor necrosis factor alpha (TNFα) signaling and steroid biosynthesis. On the other hand, 624 DEGs were uniquely regulated in isorhamnetin-treated cells, which particularly enriched transforming growth factor beta (TGFβ) and Wnt/β catenin pathways. Top 20 up and downregulated DEGs between D10 treated and control hAECs and their functions are listed in Supplementary Tables 1, 2.

### Isorhamnetin Regulated Biological Processes (BP) in D10 hAECs Compared to Undifferentiated Control (D0)

**FIGURE 2:**
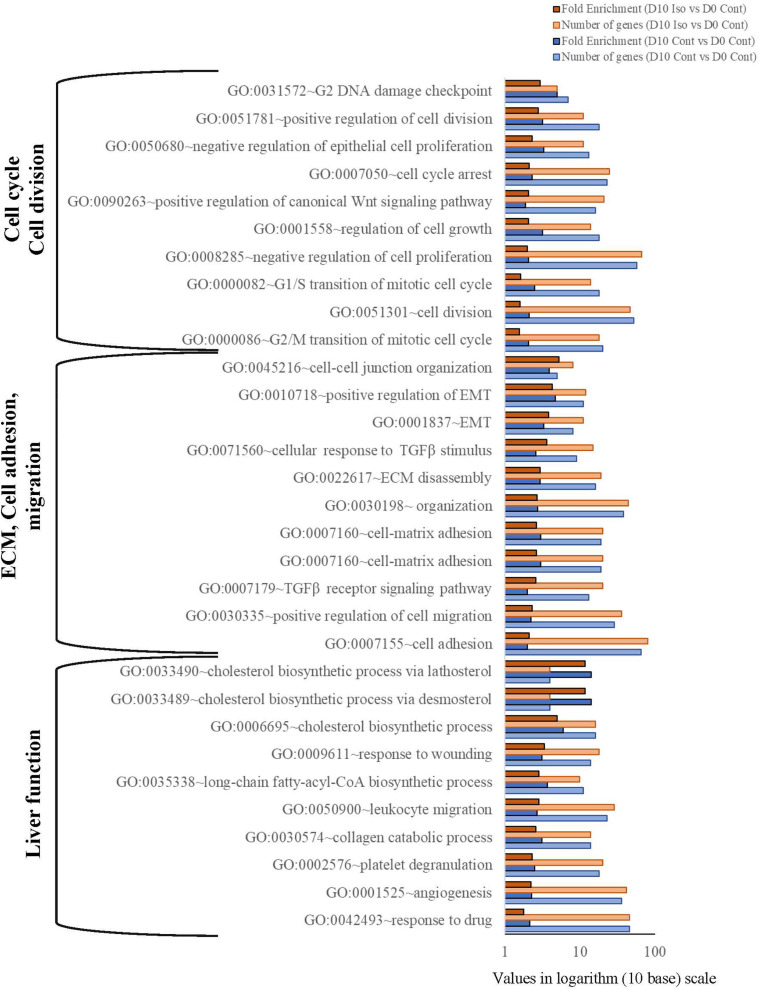
Bar graph showing significantly enriched biological process gene ontology (*p* < 0.05; Modified Fisher’s Exact Test). Values are presented in logarithm (10 base) scale. Fold enrichment is calculated as the ratio of the two proportions: the proportion of genes associated with the GO category in a list of DEGs of a study and the proportion of genes associated with that GO in the human genome (DAVID online tool: https://david.ncifcrf.gov/home.jsp). Iso, Isorhamnetin; Cont, Control.

Gene ontology analysis using DAVID’s functional analysis tool shows that several BPs related to cell cycle, extracellular matrix (ECM) organization and mature liver functions were regulated in D10 treatment and control cells (Figure 2). While cell division and cell cycle associated GOs were highly enriched in D10 control cells, cell adhesion, cell-cell junction, EMT, ECM organization, Wnt, and TGFβ signaling GOs were highly enriched in D10 isorhamnetin-treated hAECs. Furthermore, several injury stimulus-related BPs, such as platelet degranulation, angiogenesis, wound healing, were highly enriched in isorhamnetin-treated cells. The fold enrichment is calculated as the ratio of the two proportions: the proportion of genes associated with the GO category in a list of DEGs of a study and the proportion of genes associated with that GO in the human genome (DAVID online tool).

**FIGURE 3:**
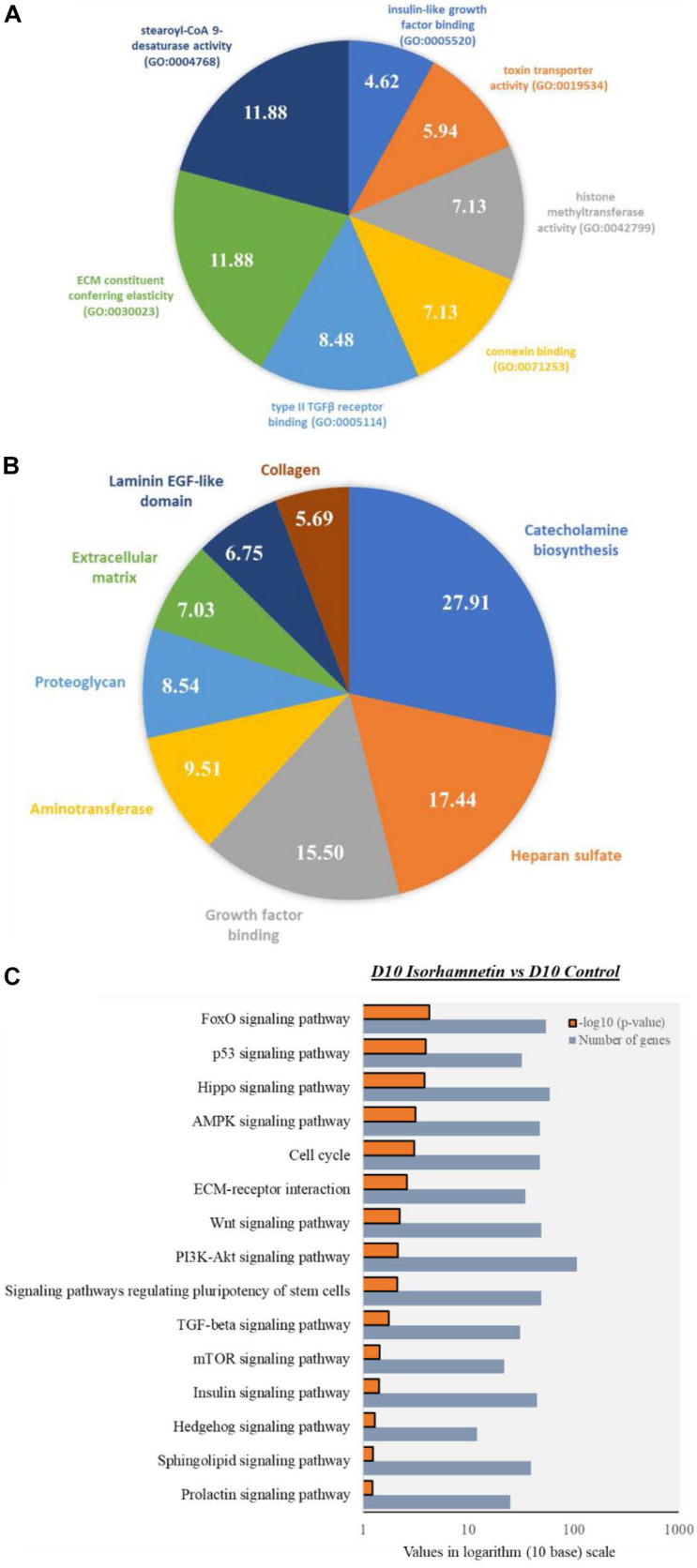
Pie charts showing top significantly enriched (*p* < 0.05; Modified Fisher’s Exact Test) **(A)** Molecular function GOs by the DEGs between D10 isorhamnetin-treated and D0 control hAECs, and **(B)** Gene functional categories of the DEGs between D10 isorhamnetin-treated and D10 control hAECs. Fold enrichment values are presented in each corresponding pie. **(C)** Bar graph showing significantly enriched (*p* < 0.05; Modified Fisher’s Exact Test) top KEGG pathways by DEGs between D10 isorhamnetin-treated and D10 control hAECs. Values are presented in logarithm (10 base) scale.

Next, we identified top enriched molecular functions (MFs) in isorhamnetin-treated cells compared to D0 control cells, which include stearoyl-CoA 9-desaturase activity (GO:0004768), ECM constituent conferring elasticity (GO:0030023), type II TGFβ receptor binding (GO:0005114), connexin binding (GO:0071253), histone methyltransferase activity (GO:0042799), and insulin-like growth factor binding (GO:0005520) (Figure 3A).

We performed gene functional classification clusters of DEGs using “Gene Functional Classification Tool” of DAVID software (Table 1). Enrichment criteria was set as: medium classification stringency; Kappa Similarity Term Overlap = 4; Kappa Similarity Threshold = 0.35; and Multiple Linkage Threshold = 0.5. Total 51 clusters were enriched by the DEGs between D10 isorhamnetin-treated hAECs and D0 control hAECs. Top enriched clusters included cell cycle, cell division, cell-adhesion, and cell junction-related GOs. DEGs between D10 isorhamnetin-treated hAECs and D10 control hAECs enriched 8 clusters, which included ECM migration, EMT, TGFβ signaling, and Wnt pathway-related GOs.

**TABLE 1:** Gene functional classification clusters of DEGs between D10 isorhamnetin-treated and D0 control hAECs and D10 isorhamnetin-treated and D10 control hAECs.

**Clusters and top enriched GO in cluster**	**Enrichment score**	**Genes in cluster**
***D10 isorhamnetin treated vs. D0 control***		
**Cluster 1** cilium assembly (GO:0060271), microtubule cytoskeleton organization (GO:0000226), mitotic cell cycle (GO:0000278), cell division (GO:0051301), protein binding (GO:0005515), microtubule binding (GO:0008017)	13.63	*SPICE1, C1orf116, CEP95, BBOF1, SPATA18, CEP162, CEP55, CEP126, MAP7D3, KIAA0753, FILIP1L, CCDC113, MISP, MTUS2, MZT2A, NREP, RAB3IP, CEP68, CAMSAP2, ZNF365*
**Cluster 2** cell division (GO:0051301), mitotic cell cycle (GO:0000278), cell population proliferation (GO:0008283), regulation of mitotic spindle organization (GO:0060236), metaphase plate congression (GO:0051310), microtubule binding (GO:0008017), protein kinase binding (GO:0019901), protein binding (GO:0005515)	9.37	*TPX2, TACC3, SPICE1, CDCA3, PRC1, MISP, MAPRE2, FAM83D, CCNG2, MAP7D3*
**Cluster 3** cell-cell adhesion (GO:0098609), cell differentiation (GO:0030154), cell junction assembly (GO:0034329), transforming growth factor beta receptor signaling pathway (GO:0007179), zinc ion binding (GO:0008270), cadherin binding involved in cell-cell adhesion (GO:0098641)	9.17	*FBLIM1, TGFB1I1, PDLIM5, ZYX, PDLIM2, AJUBA, CRIP1, ZNF185, LIMS2, C11orf54, FHL2, FHL1, LMO7, LIMA1, LMCD1, CSRP2, CSRP1, PDLIM1, TES, PRICKLE2, PXN, PDLIM7*
**Cluster 4** apical constriction (GO:0003383), regulation of cell shape (GO:0008360), integrin-mediated signaling pathway (GO:0007229), transforming growth factor beta receptor signaling pathway (GO:0007179), protein binding (GO:0005515), structural constituent of cytoskeleton (GO:0005200)	7.54	*FERMT1, RAPH1, FRMD6, FERMT2*
**Cluster 5** protein ubiquitination (GO:0016567), transcription, DNA-templated (GO:0006351)	7.51	*FILIP1L, IRF2BP2, CRBN, PRR11*

***D10 isorhamnetin treated vs. D10 control***		

**Cluster 1** sequestering of TGFβ in ECM (GO:0035583), positive regulation of cell substrate adhesion (GO:0010811), cell-matrix adhesion (GO:0007160), extracellular matrix organization (GO:0030198), calcium ion binding (GO:0005509), extracellular matrix structural constituent (GO:0005201)	7.70	*THBS3, SUSD1, EFEMP2, FBN2, FBLN2, NPNT, LTBP2, LTBP1, DNER*
**Cluster 2** O-glycan processing (GO:0016266), G protein-coupled receptor signaling pathway (GO:0007186), cell surface receptor signaling pathway (GO:0007166), cell migration (GO:0016477), cell adhesion (GO:0007155), G protein-coupled receptor activity (GO:0004930)	7.42	*LGR5, UPK3BL, TMEM40, ALPK2, LY6G6C, GPR85, MYRFL, CLEC7A, TNFRSF25, KLK5, B3GNT3, GPR1, IGFBP7, VTCN1, PSG5, MFAP3L, KERA, BAMBI, TMPRSS11A, MMP23B, SLC39A4, APCDD1, MEST, SLC1A6, CADM1, LRRC32, C1QTNF6, KCNE4, SMCO3, ADGRF5, TRBC1, ADGRF4, SEMA6D, SCARA3, SIGLEC5, B3GNT2, NTM, PAQR5, OPN3, IER3, TSPAN1, GPR155, CD248, RARRES1, IGFBPL1, ADGRF1, GALNT2, ABCG2, DNER, GALNT5, PRRG4, CSF2RB, FAM198B, AMIGO2, GPR37, SUSD1, TMPRSS11B, TMPRSS11E, ACKR3, CXADR, PTPRM, S1PR1*
**Cluster 3** cell-matrix adhesion (GO:0007160), anatomical structure morphogenesis (GO:0009653), protein binding (GO:0005515), co-receptor activity involved in Wnt signaling pathway, planar cell polarity pathway (GO:1904929)	7.25	*NTM, LYPD5, GPC4, CPM, MSLN, LY6G6C*
**Cluster 4** extracellular matrix organization (GO:0030198), collagen catabolic process (GO:0030574), extracellular matrix structural constituent (GO:0005201)	4.80	*EMILIN1, COL1A2, COL1A1, COL4A6*
**Cluster 5** positive regulation of tyrosine phosphorylation of STAT protein (GO:0042531), positive regulation of peptidyl-tyrosine phosphorylation (GO:0050731), cytokine-mediated signaling pathway (GO:0019221), cytokine receptor activity (GO:0004896)	4.09	*CSF2RB, GHR, IL6R, IL4R*

### Isorhamnetin Regulated Hepatic Differentiation Specific KEGG Pathways and Gene Functional Categories in D10 hAECs Compared to D10 Control

As GO analysis shows that both D10 control and D10 isorhamnetin-treated hAECs enriched similar BPs and MFs, we evaluated enriched functional categories of DEGs between D10 isorhamnetin-treated and D10 control cells (Figure 3B). Top enriched functional categories (according to fold enrichment) of the DEGs were catecholamine biosynthesis, heparan sulfate, growth factor binding, aminotransferase, proteoglycan, ECM, laminin EGF-like domain, and collagen.

Next, we identified significantly enriched (Modified Fisher Exact *p*-values with EASE threshold 0.1) KEGG pathways by the DEGs between D10 isorhamnetin-treated and D10 control cells (Figure 3C). Top pathways include, but not limited to, FoxO, Hippo, AMPK, Wnt, PI3K-Akt, TGFβ, mTOR, insulin, Hedgehog, Sphingolipid, and Prolactin signaling pathways, all of which are essential signaling pathways for hepatocyte differentiation.

### Isorhamnetin Did Not Regulate the mRNA Levels of Definitive Endoderm (DE) Markers

As the microarray results showed evidence of hepatic differentiation induction in isorhamnetin-treated hAECs, we further evaluated the expression of several DE markers in RT-qPCR on day 4 (D4) and D10 hAECs. However, DE markers *GATA4*, *FOXA2*, and *HNF4*α could not be detected neither in control nor in treatment cells on D4 and D10 (data not shown). Only *SOX17* was downregulated in D4 treatment and control cells compared to undifferentiated D0 cells (Supplementary Figure 3A).

### Isorhamnetin Regulated the Gene Expression of the Hepatic Progenitor (HP) Markers

**FIGURE 4:**
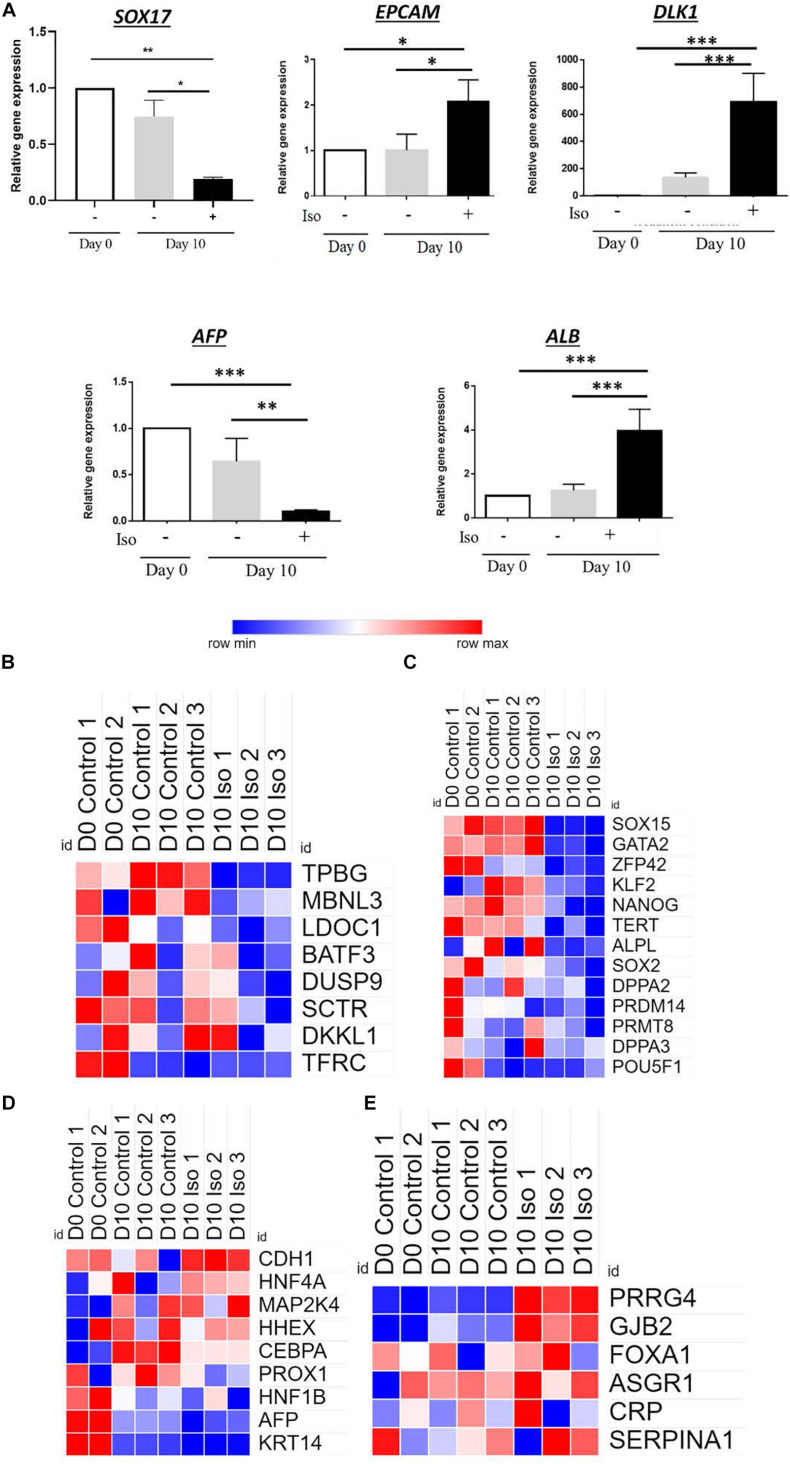
**(A)** Relative gene expression levels of hepatic progenitor markers. Heatmaps showing signal intensities (log2) of genes (from microarray analysis) related to **(B)** Undefined placenta, **(C)** Pluripotency, **(D)** Hepatoblast, and **(E)** Hepatocyte. *P < 0.05, **P < 0.01, ***P < 0.001.

Next, we evaluated the expression of several HP markers in RT-qPCR. After 10 days of treatment with isorhamnetin, delta like non-canonical notch ligand 1 (*DLK-1*) and epithelial cell adhesion molecule (EPCAM) were significantly upregulated compared to both D0 and D10 controls (Figure 4A). Also, an endoderm marker SRY-Box transcription factor 17 (*SOX17*) was significantly downregulated in isorhamnetin-treated hAECs compared to both controls. Additionally, alpha fetoprotein (*AFP*) was significantly downregulated and albumin (*ALB*) was significantly upregulated in hAECs after 10 days of isorhamnetin treatment (Figure 4A).

Furthermore, in microarray analysis, we found that the expression of genes related to undefined placenta (Figure 4B), and pluripotency (Figure 4C) were downregulated, while several hepatoblast (Figure 4D), and hepatocyte (Figure 4E) marker genes were upregulated in D10 isorhamnetin-treated hAECs.

### Isorhamnetin-Treated hAECs Did Not Retain Any Molecular Traces for Transdifferentiation and Dedifferentiation

**FIGURE 5:**
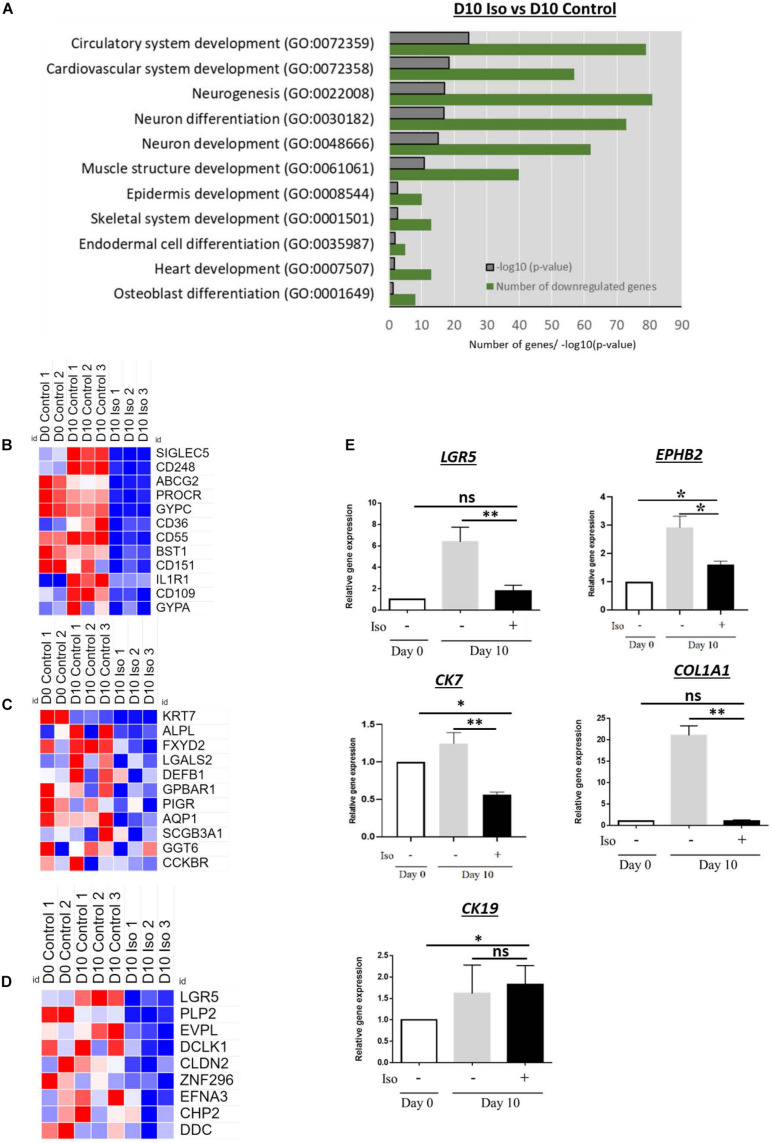
**(A)** Bar graph showing significantly downregulated GO in D10 isorhamnetin-treated hAECs compared to D10 control. Heatmaps showing signal intensities (log2) of genes (from microarray analysis) related to **(B)** Transdifferentiation, **(C)** Cholangiocyte, and **(D)** Colon. **(E)** Relative expression levels of genes to trace transdifferentiation. *P < 0.05, **P < 0.01, ns: not significant.

We evaluated whether isorhamnetin regulated the gene expressions of other cell lineages or its effect was directed exclusively to hepatocytes. In microarray, we found that several GOs related to neurogenesis, endodermal and osteoblast differentiation as well as heart and muscle structure development were significantly downregulated (Figure 5A). Heatmaps shows that the signal intensities of immune cell marker *SIGLEC5*, lung fibrocyte marker *CD248*, stem cell marker *ABCG2*, erythroid differentiation marker *GYPC* as well as markers of myeloid/osteogenic (*BST5*), chondrogenic (*CD151*), adipocyte (*CD36*), and pancreatic (*CD55*) differentiation were significantly low in isorhamnetin-treated hAECs (Figure 5B). In addition, several cholangiocyte (Figure 5C) and colon (Figure 5D)-specific markers were downregulated in isorhamnetin-treated cells.

Next, we performed the RT-qPCR to validate the microarray findings. Isorhamnetin significantly downregulated the expression of colon progenitor marker *EPHB2*, adult intestinal stem cell marker *LGR5*, cholangiocyte marker *CK7*, and fibrocyte/osteoblast lineage marker *COL1A1* (Figure 5E).

### Isorhamnetin-Treated hAECs Showed Weak CYP Enzyme Activity

**FIGURE 6:**
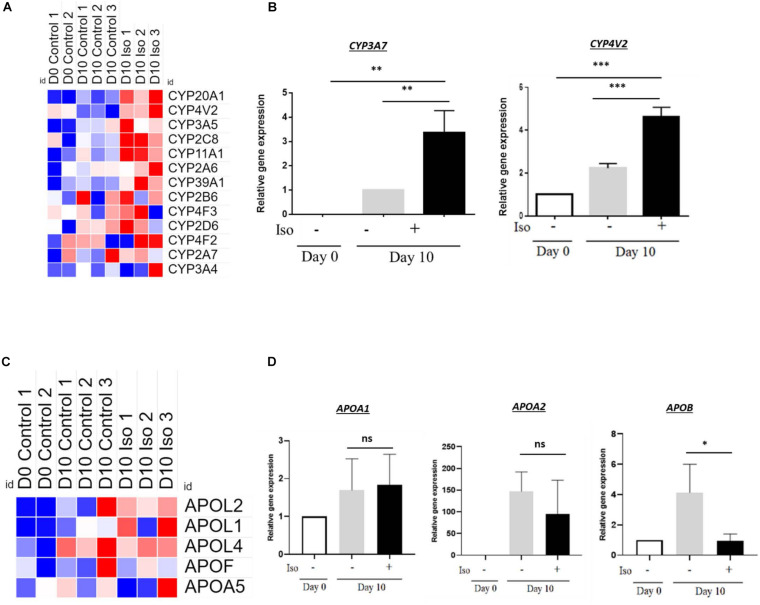
**(A)** Heatmap showing signal intensities (log2) of CYP enzymes (from microarray analysis), **(B)** Relative gene expression of CYP enzymes. **(C)** Heatmap showing signal intensities (log2) of APO genes (from microarray analysis), **(D)** Relative expression levels of APO genes. *P < 0.05, **P < 0.01, ***P < 0.001, ns: not significant.

We further investigated whether the differentiated cells in isorhamnetin have attained the characteristics of mature hepatocytes. We evaluated endogenous metabolic markers of hepatic cytochrome P450 (CYP). In microarray analysis, several CYP enzymes, namely *CYP20A1*, *CYP4V2*, *CYP3A5*, *CYP2C8*, *CYP11A1*, showed higher expression in isorhamnetin-treated hAECs compared to undifferentiated and D10 control hAECs; however, the expressions were not consistent in all experimental replicates of treatment cells (Figure 6A). Even longer treatment with isorhamnetin did not improve CYP expressions (Supplementary Figure 3B). In RT-qPCR, *CYP3A7* was undetectable on D0 and significantly upregulated in D10 isorhamnetin group compared to both controls (D0 and D10) (Figure 6B). We also checked the *CYP4V2* expression, which was also significantly upregulated by isorhamnetin treatment. However, *CYP3A4* mRNA level was undetected in all samples (D10 and D20). Similarly, no inducible CYP enzyme activity of CYP3A4 could be observed in isorhamnetin-treated hAECs (D10 and D20).

Next, we evaluated mRNA levels of lipid metabolism-related genes. Same as CYP enzymes, *APO* expressions were not consistent in all three replicates of isorhamnetin-treated hAECs (microarray results), although they showed relatively higher expressions compared to D0 and D10 controls samples (Figure 6C). In RT-qPCR, neither *APOA1* nor *APOA2* showed significant difference from D10 control (*p* > 0.05) (Figure 6D). *APOB* expression was significantly downregulated by isorhamnetin.

### Isorhamnetin-Treated hAECs Showed ICG Uptake, Glycogen Storage, and Urea Production

**FIGURE 7:**
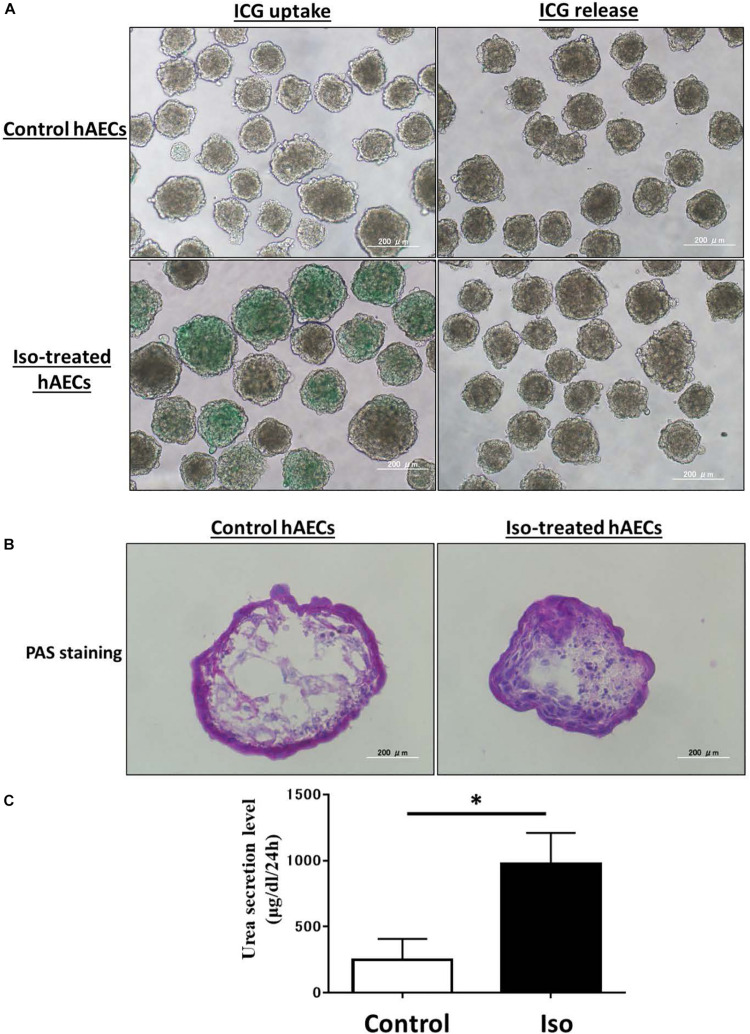
**(A)** ICG uptake and release, **(B)** PAS staining, **(C)** Urea secretion assay. *P < 0.05.

Finally, we assessed the ability of isorhamnetin-treated differentiated hAECs for mature hepatocyte functions. ICG is a cyanine dye that can be uptaken and subsequently cleared by hepatocytes. The ICG uptake and clearance test is used for a rapid assessment of liver function in clinical settings. We found that isorhamnetin-treated hAECs showed ICG uptake and clearance after 6 h; conversely, untreated D10 control cells did show very marginal/undetectable ICG uptake and clearance (Figure 7A).

Moreover, the liver has the ability to store glycogen. PAS can stain glycogen. Isorhamnetin-treated hAECs showed PAS stained cells, which indicated hAECs could store glycogen (Figure 7B). In a previously published article, it was shown that untreated hAECs could also exhibit PAS staining (Furuya et al., 2019). Similarly, in our study we found that untreated control group also had PAS stained cells.

Additionally, we measured urea and albumin secretion activities in isorhamnetin-treated hAECs. Treated hAECs secreted significantly higher urea compared to untreated control cells (Figure 7C). However, ALB secretion could not be detected in isorhamnetin-treated cells on the protein level on D10 and D20.

## Discussion

In the present study, we have reported for the first time the potential role of a naturally occurring compound isorhamnetin in inducing hepatic-lineage-specific differentiation in hAECs maintained in 3D culture conditions in the absence of any other growth factor. We used whole-genome transcript analysis to study early biological events that regulated controlled differentiation in isorhamnetin-treated hAECs. After 10 days of treatment with isorhamnetin, the hAECs expressed a subset of hepatic differentiation-related genes, induced CYP mRNA levels, showed ICG uptake and release, stored glycogen, and secreted urea. However, the differentiated cells could not exert some key features of hepatic maturation, such as ALB secretion and CYP enzyme activity.

In the present study, we used hAECs that were isolated from the full-term placenta. The cells were preserved at the Tsukuba Human Tissue Biobank Center (THB) established at the University of Tsukuba Hospital (Takeuchi et al., 2016). The primary amnion epithelial cells were heterogenous; however, hAECs isolated from the adherent subpopulations of passaged primary cells and cultured in 3D environment showed homogeneous characteristics (Furuya et al., 2019).

Isorhamnetin is an *O*-methylated flavonol occurring naturally in plants but is also a methylated metabolite of quercetin (Magar and Sohng, 2020). Studies have reported that isorhamnetin exerts an anti-inflammatory effect in murine RAW264.7 cells (Boesch-Saadatmandi et al., 2011), inhibits the proliferation of breast cancer cells (Hu et al., 2015), protects HepG2 cells against oxidative stress (Yang et al., 2014), attenuates inflammatory bowel disease (Dou et al., 2014), and represses adipogenesis in 3T3-L1 cells (Lee et al., 2009). We have previously shown that isorhamnetin has antioxidant, antiobesity, and antifibrotic effects in rodent models (Zar Kalai et al., 2013; Ganbold et al., 2019). Isorhamnetin exerts its bioactivities through regulating Wnt upstream to β catenin in colorectal cancer cells (Park and Choi, 2010; Amado et al., 2011). Besides the effect on tumor cells, isorhamnetin may also function as a modulator of the Wnt pathway in other cell types. Also, isorhamnetin attenuates liver fibrosis by inhibiting TGFβ signaling pathways in hepatic stellate cells (Yang et al., 2016). Both Wnt/β catenin and TGFβ pathways are implicated in almost every facet of liver biology- from liver cell fate decision to liver organogenesis and to liver pathologies (Clotman et al., 2005; Clotman and Lemaigre, 2006; Nejak-Bowen and Monga, 2008; Touboul et al., 2016; Russell and Monga, 2018). We found that Wnt/β catenin and TGFβ pathways were highly enriched in isorhamnetin-treated hAECs, which may induce directed differentiation in hAECs.

Additionally, several other hepatic differentiation-related KEGG pathways were also enriched. Hedgehog Signaling is implicated in the proliferation of a liver stem cell subgroup named small hepatocyte-like progenitor cells as well as in end-stage liver diseases through orchestrating the wound healing process (Wang et al., 2016; Machado and Diehl, 2018). The Hippo signaling pathway controls liver cell fate and influences liver regeneration (Yimlamai et al., 2014; Manmadhan and Ehmer, 2019). FoxO signaling is considered as the metabolic regulator of the liver because of its involvement in hepatic gluconeogenesis and lipid metabolism (Shin et al., 2012; Tikhanovich et al., 2013). This pathway is reciprocally regulated by insulin through PI3K/Akt pathway. Among other enriched pathways, the mTOR pathway is involved in liver regeneration and autophagy functions (Fouraschen et al., 2013), and Sphingosine 1-Phosphate Signaling pathway in liver fibrosis through regulating pleiotropic cell responses to inflammation, such as cell survival, migration, and vascular permeability (González-Fernández et al., 2017). In addition, Prolactin signaling not only regulates postnatal liver growth in rodents (Moreno-Carranza et al., 2018) but also regulates liver regeneration by stimulating hepatocyte proliferation, promoting angiogenesis, downregulating IL-6, and upregulating SOCS3 (Moreno-Carranza et al., 2013).

Gene ontology analysis showed that 10 days treatment with isorhamnetin could significantly activate both early and late phase BPs involved in the induction of hepatic differentiation, including cell division, cell migration, cell adhesion, tight junction, EMT, ECM organization as well as cholesterol and fatty acid biosynthesis, angiogenesis, and wound healing.

Top enriched MFs showed isorhamnetin had histone methylation activity, which has been reported to enhance hepatic regeneration (Zlotorynski, 2019). Stearoyl-CoA desaturase activity has also been reported to modulate hepatocyte differentiation from human iPSCs (Rahimi et al., 2015). Also, connexin-mediated gap junctional intercellular communication is reported to modulate stepwise hepatic lineage restriction and maturation from ESCs (Qin et al., 2016).

Functional categories of downregulated DEGs included aminotransferase and collagen. Aminotransferases are the most common liver injury markers (McGill, 2016), whereas collagen induces liver fibrosis (McGill, 2016). Laminins, basement membrane protein family, have been implicated in the maintenance of hepatic differentiation (Kikkawa et al., 2011). Heparan sulfate proteoglycans are essential cofactors in receptor-growth factor interactions, as well as in cell-matrix adhesion and have been implicated in both normal and diseased liver conditions (Roskams et al., 1995; Tátrai et al., 2010).

Hepatic differentiation from stem cells is widely studied to apply for regenerative therapy and drug screening tests. It is already elucidated that the differentiation process for hepatocyte follows DE, progenitor, and then, hepatocyte. And during a certain stage, some genes are strongly and specifically expressed. In our study, we found that isorhamnetin downregulated the expression of DE marker *SOX17*; however, other DE markers, *GATA4*, *FOXA2*, and HP marker *HNF4*α were not regulated. Previously, Marongiu et al. (2011) reported that hAECs pretreated with Activin A for 4 days for inducing endodermal commitment did not express FOXA2 and SOX17, whereas Liu et al. (2018) reported that hAECs cultured in endodermal induction medium in the presence of EGF and bFGF for 2 days expressed both Sox17 and FOXA2. However, neither of the studies reported HNF4α expression status. mRNA levels of other HP markers, *DLK-1* and *EPCAM* were significantly upregulated by isorhamnetin treatment. As shown before, the expression level of *AFP* was decreased, while *ALB* was increased in our study. It has been reported that AFP is strongly expressed in the progenitor phase, and then, AFP should be gently downregulated with the progression of hepatic differentiation. Oppositely, ALB expression increases with the progression of differentiation (Chaudhari et al., 2016). However, ALB secretion could not be detected on protein level on D10 and D20 isorhamnetin treatment despite significantly higher mRNA expression level on D10 isorhamnetin-treated hAECs. Previous study reported that ALB protein level was only detected after culturing hAECs in hepatic maturation medium containing EGF, Oncostatin M, HGF, dexamethasone, etc. (Liu et al., 2018).

We further evaluated several hepatic functions in the isorhamnetin-treated hAECs. CYP is a superfamily enzyme that catalyzes many exogenous compounds and endogenous oxidations. And some CYP enzymes, including CYP1A2 and CYP3A4, catalyze drugs in the human liver (Rodríguez-Antona et al., 2001). *CYP3A7* is produced in fetal liver; conversely, 3A4 is in the adult liver (Lacroix et al., 1997). Thus, the expression of these markers is essential for the functional hepatocytes and can be used to trace cell fate. We found that isorhamnetin significantly upregulated *CYP3A7*; however, *CYP3A4* could not be detected. Even more prolonged treatment with isorhamnetin (day 20) neither expressed *CYP3A4* nor showed its inducible enzyme activity. Previously, it was reported that hAECs cultured in a hepatogenic induction medium containing EGF, bFGF, HGF, and dexamethasone had low expression of mature hepatocyte marker CYP3A4 (Liu et al., 2018). Lin et al. (2015) also showed that hepatic differentiated AECs following a four-step hepatic differentiation protocol did not show any significant change in CYP3A4 mRNA levels. Additionally, hepatic lipid metabolism genes, *APOA1* and *APOA2*, were also not regulated by isorhamnetin treatment on D10. Isorhamnetin-treated hAECs showed ICG uptake and release, glycogen storage, and urea secretion activities.

Altogether, our findings suggest that isorhamnetin could induce hepatic-lineage specific targeted differentiation in hAEC. Although the differentiated cells showed some functional characteristics of hepatocytes, they failed to achieve some key features such as ALB secretion and CYP enzyme activities.

Natural compounds with proven bioactivities can control cellular behavior and early biological and molecular events to direct the targeted differentiation of stem cells in a lineage-specific manner. Only a handful of studies were conducted to evaluate the effects of naturally occurring compounds on hAECs (Ferdousi et al., 2019, 2020; Hou et al., 2020). Furthermore, while current researches focus on the safety and efficacy of translating hAEC therapy into clinical practices (Murphy et al., 2010; Strom et al., 2013; Lim et al., 2017, 2018; Srinivasan et al., 2020), priming approaches using natural bioactive compounds to improve its effectiveness has not been explored. In the present study, as we could demonstrate that isorhamnetin, a methylated flavonol, induced hepatic-lineage specific differentiation in hAECs, other flavonols with similar chemical structure and functionality may show similar or better differentiation-inducing effects on hAECs.

In conclusion, our study is the first to report that a naturally occurring compound isorhamnetin could induce hepatic-lineage-specific differentiation in hAECs without any additional growth factor or cytokine. However, hepatic cell maturation could not be obtained even after treatment with isorhamnetin for a longer duration. Therefore, further research is warranted to explore potential bioactive compounds, and optimal culture condition and microenvironment to attain functional hepatocytes from hAECs and finally to evaluate the differentiated cells in acute liver disease *in vivo* models.

## Data Availability Statement

The datasets presented in this study can be found in online repositories. The names of the repository/repositories and accession number(s) can be found in the article/Supplementary Material. Microarray data are deposited in the Gene Expression Omnibus (GEO) under accession number GSE148777 (https://www.ncbi.nlm.nih.gov/geo/query/acc.cgi?acc=GSE148777).

## Ethics Statement

The Ethical Review Committee of the University of Tsukuba Hospital approved the protocol. Informed written consent was received from the mothers who donated the placenta after delivery.

## Author Contributions

YU: investigation, data curation, formal analysis, visualization, and writing – original draft. FF: conceptualization, methodology, formal analysis, visualization, and writing – original draft. Y-WZ: conceptualization, methodology, funding acquisition, project administration, and supervision. TO: conceptualization, resources, and funding acquisition. HI: conceptualization, methodology, funding acquisition, project administration, resources, supervision, and writing – review and editing. All authors made substantial contributions to this article and approved the final article.

## Conflict of Interest

The authors declare that the research was conducted in the absence of any commercial or financial relationships that could be construed as a potential conflict of interest.

## Footnotes

1 https://www.ncbi.nlm.nih.gov/geo/query/acc.cgi?acc=GSE148777

2 http://www.affymetrix.com

3 https://software.broadinstitute.org/gsea/index.jsp

4 https://software.broadinstitute.org/morpheus

5 http://bioinformatics.psb.ugent.be/webtools/Venn/

## Abbreviations

AFP: Alpha-fetoprotein
ALB: Albumin
BMP: Bone morphogenetic protein
COL1A1: Collagen type 1 alpha 1
CYP3A4: Cytochrome P450 3A4
CYP3A7: Cytochrome P450 3A7
DAVID: Database for Annotation Visualization and Integrated Discovery
DLK-1: Delta-like-1
EGF: Epidermal growth factor
EGFR: Epidermal growth factor receptor
EPCAM: Epithelial Cell Adhesion Molecule
ESC: Embryonic stem cell
FGF: Fibroblast growth factor
hAEC: Human amniotic epithelial cell
HHEX: Hematopoietically expressed homeobox
HLC: Hepatocyte-like cells
HNF4a: Hepatocyte nuclear factor 4 alpha
ICG: Indocyanine Green
iPSC: Induced pluripotent stem cell
Klf4: Kruppel Like Factor
LGR5: Leucine-rich-repeat-containing G-protein -coupled receptor 5
NANOG: Homeobox protein NANOG
OCT4: Octamer-binding transcription factor 4
PAS: Periodic Acid Schiff
TGF- β: Transforming growth factor- β
