# Supplementary material for: Global Gene Expression Profiling Reveals Isorhamnetin Induces Hepatic-Lineage Specific Differentiation in Human Amniotic Epithelial Cells
Source: Front Cell Dev Biol. 2020 Nov 5;8:578036. doi: 10.3389/fcell.2020.578036 (PMC7674172; doi:10.3389/fcell.2020.578036)
Supplement: Supplementary file 1 [file Data_Sheet_1.PDF]

Supplementary Material

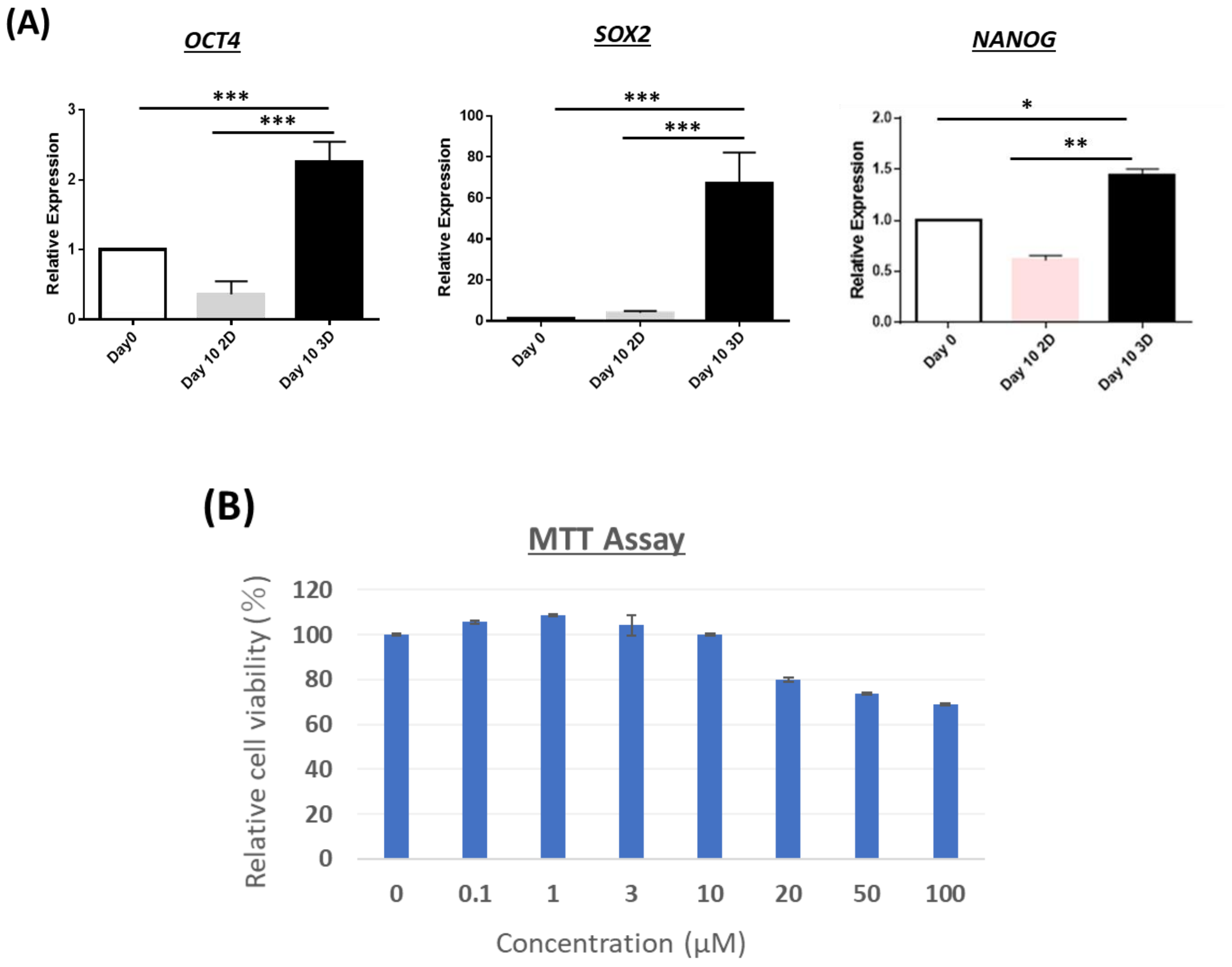

Supplementary Figure 1. (A) Relative expression of stemness markers in 3D hAECs. **(B)** MTT assay.

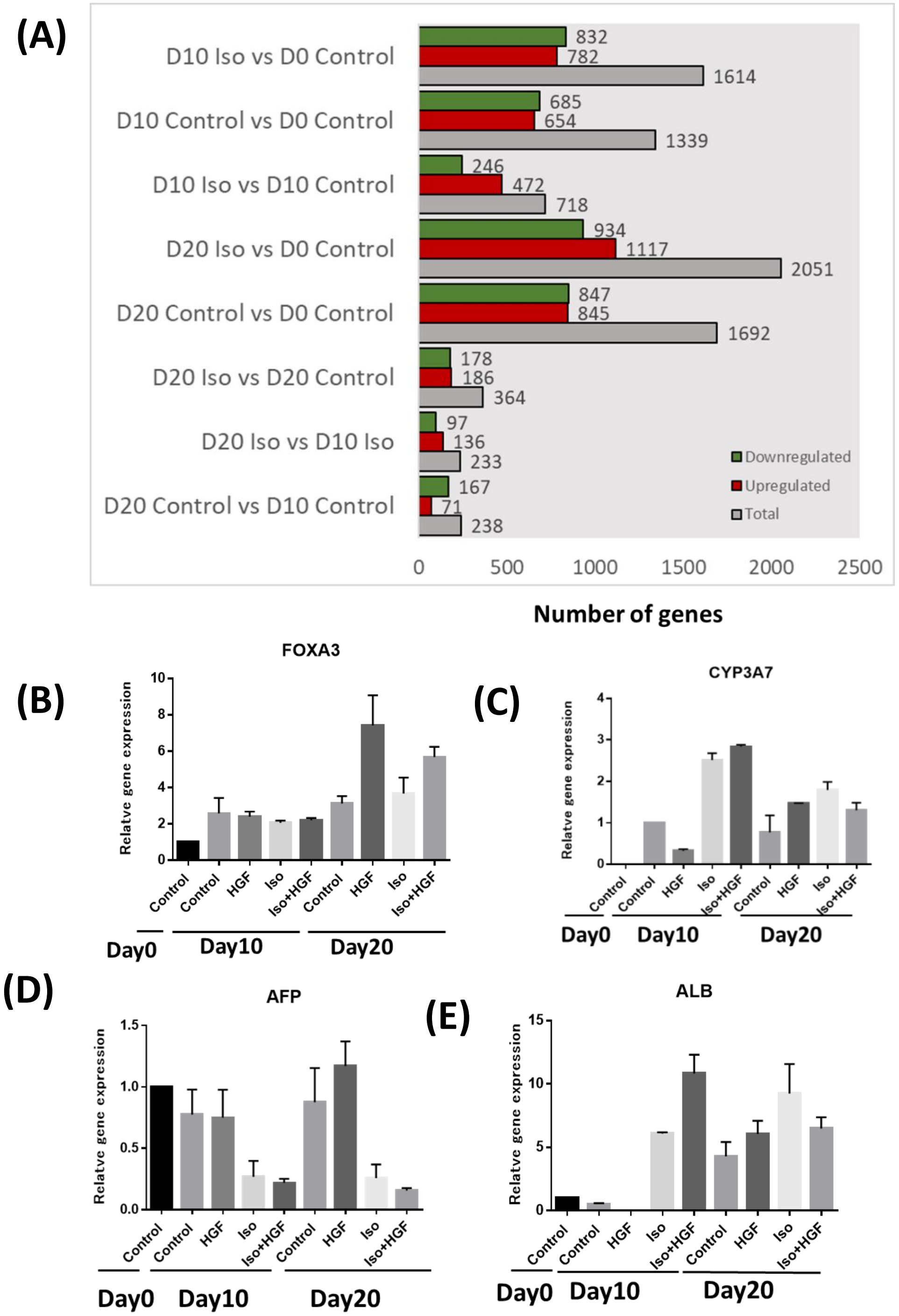

Supplementary Figure 2. (A) Bar graph showing number of DEGs in each treatment pair. **(B-E)** Relative gene expression up to day 20 isorhamnetin treatment.

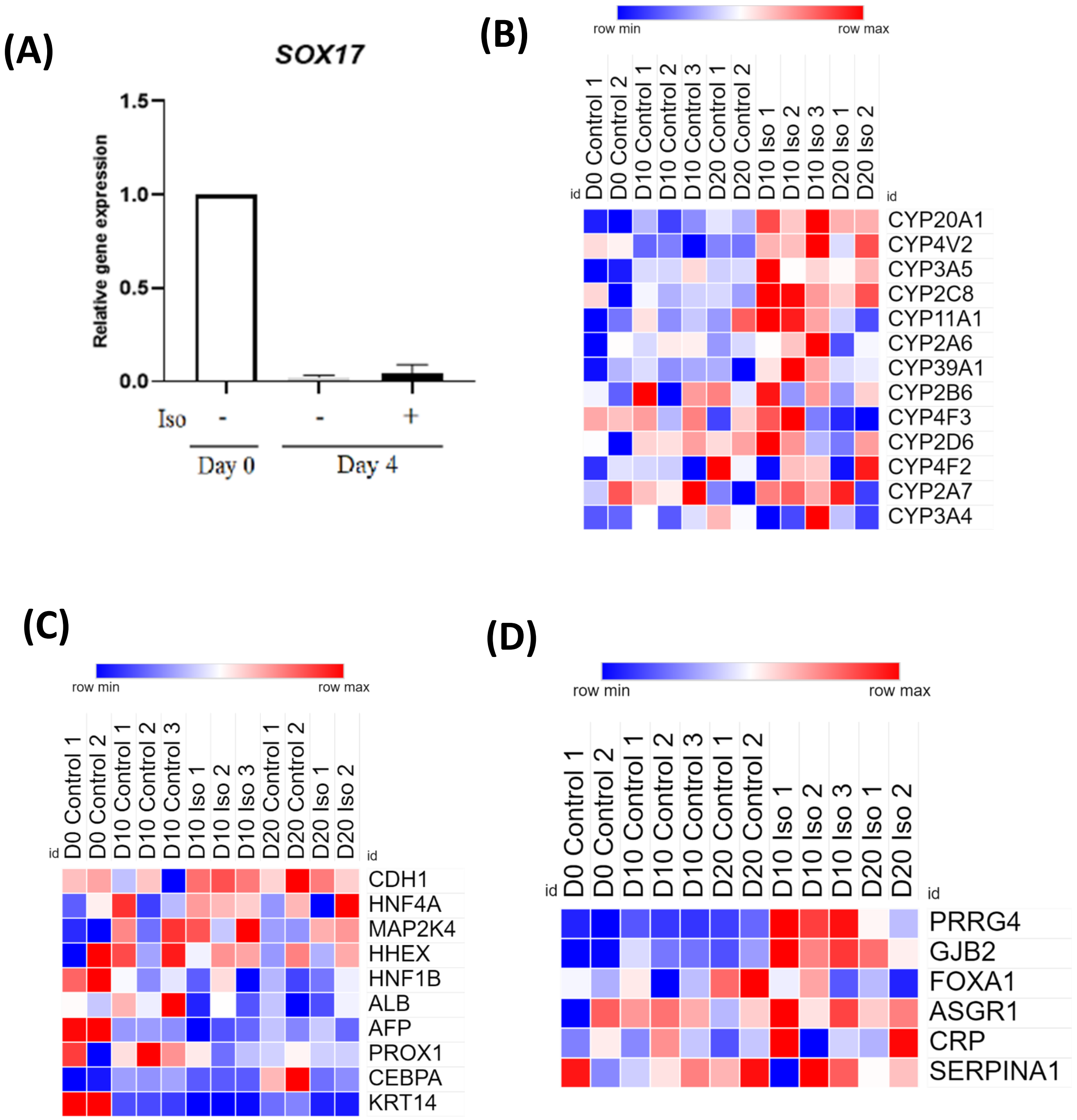

Supplementary Figure 3. (A) Relative expression of *SOX17* on day 4. Heatmaps showing gene expression intensities of **(B)** CYP enzymes, **(C)** Hepatoblast markers, **(D)** Hepatocyte markers

Supplementary Table 1. Top 20 upregulated DEGs between D10 isorhamnetin-treated and D10 control hAECs and their function

Gene Symbol	Description	Fold Change	P-value	Function
SFRP2	secreted frizzled-related protein 2	6.93	6.74E-13	modulators of Wnt signaling through direct interaction with Wnts. They have a role in regulating cell growth and differentiation in specific cell types
CLIC5	chloride intracellular channel 5	6.47	7.74E-11	regulation of ion transmembrane transport
NPNT	nephronectin	6.08	5.37E-13	Functional ligand of integrin alpha-8/beta-1, regulating cell adhesion
MYCN	v-myc avian myelocytomatosis viral oncogene neuroblastoma derived homolog	6.02	5.66E-13	DNA-binding transcription factor activity
PCDH20	protocadherin 20	6.0	5.26E-12	Potential calcium-dependent cell-adhesion protein
GPAT3	glycerol-3-phosphate acyltransferase 3	5.83	2.84E-10	regulation of TOR signaling, triglyceride biosynthetic process
CLDN3	claudin 3	4.84	6.22E-12	tight junction, cell-adhesion
SEMA6D	sema domain, transmembrane domain (TM), and cytoplasmic domain, (semaphorin) 6D	3.49	3.16E-10	positive regulation of cell migration
KRT13	keratin 13, type I	3.38	1.01E-10	cytoskeleton organization
LRRC32	leucine rich repeat containing 32	3.3	4.12E-11	Key regulator of transforming growth factor beta (TGFB1, TGFB2 and TGFB3) that controls TGF-beta activation
KERA	keratocan	3.3	1.47E-09	response to stimulus
ALDH1A2	aldehyde dehydrogenase 1 family, member A2	3.28	8.47E-11	liver development
FAT4	FAT atypical cadherin 4	3.27	8.64E-09	calcium-dependent cell adhesion proteins

SLC40A1	solute carrier family 40 (iron-regulated transporter), member 1	3.26	4.81E-11	cellular iron ion homeostasis
FAM102B	family with sequence similarity 102, member B	3.25	7.28E-09	alternative splicing
S1PR1	sphingosine-1-phosphate receptor 1	2.83	1.07E-09	G protein-coupled receptor activity, angiogenesis, cell adhesion
TMEM38B	transmembrane protein 38B	2.75	4.46E-08	extracellular matrix constituent secretion
MEST	mesoderm specific transcript	2.72	1.40E-09	regulation of lipid storage
PMP22	peripheral myelin protein 22	2.66	2.49E-09	negative regulation of cell population proliferation
RGS4	regulator of G-protein signaling 4	2.65	5.21E-09	G protein-coupled receptor signaling pathway

Supplementary Table 2: Top 20 downregulated genes between D10 isorhamnetin-treated and D10 control hAECs and their function

Gene Symbol	Description	Fold Change	P-value	Function
CYP1B1	cytochrome P450, family 1, subfamily B, polypeptide 1	-22.5	9.05E-12	metabolism of various endogenous substrates, including fatty acids, steroid hormones and vitamins
DKK2	dickkopf WNT signaling pathway inhibitor 2	-10.5	2.96E-12	Antagonizes canonical Wnt signaling by inhibiting LRP5/6 interaction with Wnt
ADGRF1	adhesion G protein-coupled receptor F1	-10.44	5.48E-13	cell surface receptor signaling pathway
COL1A1	collagen, type I, alpha 1	-10.03	8.48E-13	extracellular matrix structural constituent
C5orf46	chromosome 5 open reading frame 46	-9.23	6.94E-14	Uncharacterized
THBS1	thrombospondin 1	-8.55	2.53E-11	activation of MAPK activity, laminin binding
COL1A2	collagen, type I, alpha 2	-7.66	3.69E-13	extracellular matrix structural constituent
LGR5	leucine-rich repeat containing G protein-coupled receptor 5	-7.33	1.37E-12	marker of adult tissue stem cells in the intestine, stomach, hair follicle, and mammary epithelium
CRIP1	cysteine-rich protein 1 (intestinal)	-6.5	3.86E-12	function as an intracellular zinc transport protein
ODAM	odontogenic, ameloblast asssociated	-6.05	1.98E-11	inflammatory response
FAP	fibroblast activation protein alpha	-5.9	1.32E-10	participates in extracellular matrix degradation and involved in many cellular processes including tissue remodeling, fibrosis, wound healing, and inflammation
MAMDC2	MAM domain containing 2	-5.89	4.17E-11	Extracellular matrix component
GNA14	guanine nucleotide binding protein (G protein), alpha 14	-5.74	2.32E-11	modulators in various transmembrane signaling systems
CLCA2	chloride channel accessory 2	-5.63	6.33E-12	cell adhesion
RYR3	ryanodine receptor 3	-5.55	2.50E-08	cellular calcium signaling
FILIP1L	filamin A interacting protein 1-like	-5.44	1.39E-11	inhibition of cell proliferation and migration, antiangiogenic activity

MGAM	maltase-glucoamylase	-5.43	2.39E-13	amylase activity
KCNE4	potassium channel, voltage gated subfamily E regulatory beta subunit 4	-5.36	1.01E-12	voltage-gated potassium channel activity
ENDOU	endonuclease, polyU-specific	-5.01	3.75E-11	immune response, growth factor activity
TPRG1	tumor protein p63 regulated 1	-4.96	6.12E-12	Cellular component of cytoplasm
